# Capability Analysis of AZ91D Magnesium Alloy Precision Milling Process with Coated Tools

**DOI:** 10.3390/ma16083119

**Published:** 2023-04-15

**Authors:** Jarosław Korpysa, Józef Kuczmaszewski, Ireneusz Zagórski

**Affiliations:** Department of Production Engineering, Faculty of Mechanical Engineering, Lublin University of Technology, Nadbystrzycka 36, 20-618 Lublin, Poland; j.kuczmaszewski@pollub.pl (J.K.); i.zagorski@pollub.pl (I.Z.)

**Keywords:** AZ91D magnesium alloy, precision milling, process capability

## Abstract

Process capability analysis is the main tool of statistical process control. It is used for the ongoing monitoring of product compliance with imposed requirements. The main objective and novelty of the study were to determine the capability indices for a precision milling process of AZ91D magnesium alloy. Machining was performed in terms of variable technological parameters and using end mills with protective TiAlN and TiB_2_ coatings intended for the machining of light metal alloys. The Pp and Ppk process capability indices were determined based on the measurements of the dimensional accuracy of the shaped components that were taken on a machining centre with a workpiece touch probe. Obtained results demonstrated that the type of tool coating and variable machining conditions had a significant impact on the machining effect. The selection of appropriate machining conditions enabled a terrific level of capability to be achieved at a tolerance of 12 µm, several times lower than under unfavourable conditions where the tolerance was up to 120 µm. Improvements in process capability are mainly achieved by adjusting the cutting speed and feed per tooth. It was also shown that process estimation based on improperly selected capability indices might lead to an overestimation of the actual process capability.

## 1. Introduction

Process capability is one of the issues analysed in statistical process control (SPC). This method is used to manage the quality of products and manufacturing processes. It is primarily employed to keep process effects within the acceptable lower specification limit (LSL) and upper specification limit (USL), which are requirements imposed by the client. The difference between the upper and lower specification limits is a tolerance range T that should encompass obtained results [1,2,3].

Process capability can be estimated based on pre-defined quality indices. One can distinguish indices for estimating short-term process capability (Cp, Cpk) and long-term process capability (Pp, Ppk), which are also called process performance indices. These indices always come in pairs—the first one defines the potential capability of a process, while the other one (with an index k) defines the degree of process centring and provides information about its real capability. Both types of indices are estimated almost identically, the only difference being the way in which standard deviation is determined. In the case of the Cp and Cpk indices, the process variability is determined in relation to individual measurement groups, whereas with the Pp and Ppk indices, overall variability is estimated for the entire process. The way of interpreting results is also the same for both groups of indices. The higher the index value is, the better the process capability becomes. The most widespread index levels used in industry are as follows: 1—fair, 1.33—good, 1.67—excellent, and 2—terrific [2,3,4]. Although the generally accepted level is 1.33, the value depends on the nature of a given manufacturing process. For example, the largest companies from the automotive sector, where product quality is fundamental, take a value of 1.67 or even 2 as the minimal value [1,5]. Naturally, these are only conventional thresholds, and companies can adjust their values to suit individual needs. Nonetheless, the proposed values follow directly from the properties of the normal distribution to which analysed data should be subjected and the 6-sigma principle [6,7]. The range of ±3σ from the mean value contains about 99.73% of all results; hence for the capability index equal to 1, the defect rate is 0.27% of products. The defect rate decreases with increasing process capability. By narrowing the process variability to 50% of the assumed tolerance range (the capability index equal to 2), it is possible to reduce the defect formation risk to 0.002 ppm (parts per million) [5,8]. However, the selection of the desired process capability level should also depend on economic aspects [9,10]. Process capability indices refer to only one variable, while multiple product properties are usually controlled. The total probability of product compliance is, therefore, the quotient of indices for all monitored variables. A number of variables can also be estimated based on specifically designed indices [5,11,12].

Statistical process control is one of the basic tools for monitoring manufacturing processes. It can be successfully employed in many industries and applied to various manufacturing processes. This has been confirmed using research studies on the control of various reductive machining methods such as milling, turning, drilling, and grinding [4,13,14]. All manufacturing processes are burdened with errors, which is why process control is so important. In milling, part accuracy depends on, among other things, the accuracy of the process itself applied to machines and tools as well as machining conditions [7]. The human factor is also significant and can impact the effects of the process as well as measurement results [15]. As the complexity of the milling process and the requirements for manufactured products increase, it is necessary to control a growing number of factors affecting the milling process and its end result, e.g., tool wear progressing during machining [8,16]. For this reason, modern production lines are equipped with an increasing number of sensors designed to ensure and maintain the highest possible process capability [7].

Process capability indices can also be used to determine optimal machining conditions in terms of product quality as well as process efficiency. This pertains to both attempts aimed at increasing process capability in order to achieve the required level or—less frequently—to reduce it. It is unjustified to maintain the process capability indices well above the required values. While this may reduce the likelihood of producing defective parts, it is also usually associated with generating an unnecessary increase in costs [17,18]. The key feature which is subjected to control in manufacturing processes is dimensional and geometrical accuracy. It is the basic parameter defining product quality, as it determines the cooperation of two or more mating parts. The maintenance of specified tolerances and fits is, hence, particularly important. Dimensional accuracy control is also important when it comes to components susceptible to dimensional change or deformation, such as thin-walled parts [19,20].

A capability analysis was performed in [18] for Inconel 718 micromilling. The process capability was determined based on measurements of the width of the microchannels. Both dry and MQL milling resulted in insufficient process capability, as indicated by the low indices of Cp = 0.17 and Cp = 0.36, respectively. Lin et al. [21] analysed the micromilling of printed circuit boards (PCBs) made of compound materials. Initially, higher values of the indices Cp = 1.67 and Cpk = 1.06 were obtained for the TiAlCN-coated tool compared to the uncoated tool—Cp = 1.36 and Cpk = 0.79. However, when offset improvement was applied, the higher capability was obtained with the uncoated end mill (Cp = 3.57; Cpk = 3.17) than with the TiAlCN-coated end mill (Cp = 2.50; Cpk = 2.42). The research has been continued in [22], with the use of uncoated and ZrN-coated tools this time. The study showed that a higher process capability was obtained using the coated tool (Cp = 1.99; Cpk = 1.86) against the uncoated tool (Cp = 1.36; Cpk = 0.79). This is associated with reduced tool wear, affecting dimensional stability. An attempt to optimise technological parameters during slot milling in aluminium was reported in [23]. The results indicated that an appropriate selection allows a significant improvement in process capability. Cp values increased from 0.05 to 2.75 for the first slot and from 0.06 to 2.86 for the second slot.

In addition to dimensional accuracy control, the process capability indices can also be successfully used to monitor other measurable quantities, such as surface roughness. In the study [24], process capability indices were used to determine the milling conditions that provided the best surface quality. An uncoated carbide end mill and a TiN-coated end mill were used to machine 42CrMo4 steel. Through changing the technological parameters, the value of the Cp index was increased from 1.22 to 6.16 for the uncoated tool and from 2.86 to 15.70 for the coated tool. The use of process capability indices for roughness control is also described in [25]. X6CrNiTi18-10 steel surfaces were shaped using an abrasive water jet (AWJ). The process capability in the first and second cutting zones was very good, while in the third zone, the results indicated that the process was not centred. The surface roughness also increased as the cutting speed increased. Statistical process control is thus highly universal and can be used to produce components that must meet stringent requirements. Therefore, it is justified that the process capability indices be applied to evaluate precision machining, where high accuracy and manufacturing quality are of the utmost importance [26,27].

The novelty of the presented study is mainly the analysis of the influence of machining conditions on milling process capability. Despite the fairly widespread use of statistical process control tools in industry, the number of publications in this field is negligible, as confirmed by the presented literature review. The analysis of the results obtained in terms of the dimensional accuracy of workpieces will especially allow the results to be considered from a more practical and industrial point of view. An additional innovation of the conducted research is the realisation of the magnesium alloy milling process under precision machining conditions. Although precision milling is a machining method that has been known for a long time, its application to magnesium alloy machining has so far only been described in a few papers. It is, therefore, necessary to better understand and develop precision machining for this group of materials.

## 2. Materials and Methods

The study described in this paper involved conducting precision milling tests and then measuring dimensional accuracy and determining values of the process capability indices.

The milling tests were conducted on the specimens of AZ91D magnesium alloy. This casting alloy is one of the most widely used magnesium alloys. Magnesium alloys have the lowest density among the available constructional materials, and as far as it is possible and cost-effective, they are replacing commonly used steel or aluminium alloys. Their use is quite widespread in the automotive, aerospace and medical industries. The chemical composition of the material used in the research is shown in Table 1.

Two end mills with a diameter of 16 mm and three cutting edges were used in the tests. The cutting part of the tools was coated with protective coatings made of TiAlN and TiB_2_. Both tools, as well as tool coatings, were dedicated to the machining of light metal alloys. The tools mounted in shrink-fit tool holders were balanced to G2.5 class at 25,000 rpm, in compliance with ISO 21940-1 [28]. The milling tests were carried out on the AVIA VMC 800HS (Warsaw, Poland) vertical milling centre, having an electro-spindle with a maximum speed of 24,000 rpm to enable high-speed machining.

The basic technological parameters: a cutting speed v_c_, a feed per tooth f_z_, and an axial depth of cut a_p_ were variable, while a radial depth of cut a_e_ was maintained constant at 14 mm. A plan of experiments conducted with the use of both tools is shown in Table 2.

**Figure 1:**
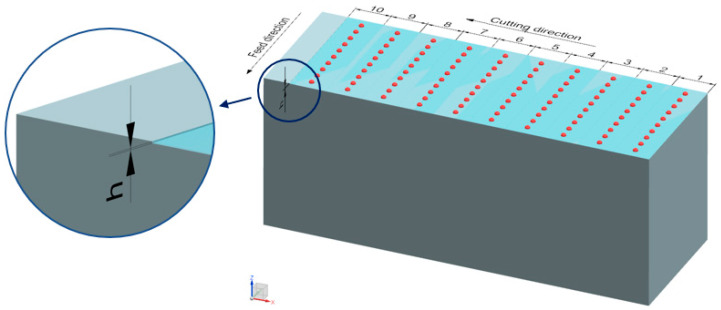
Model of the milling process and dimensional accuracy measurement [27].

Immediately after the milling process, obtained parts were measured for their dimensional accuracy. To avoid errors associated with the change of mounting of the workpiece, measurements were taken on the machine tool with a TS 640 Heidenhain (Traunreut, Germany) workpiece touch probe. Measurements were taken in the *Z*-axis of the machine tool, as shown in the diagram in Figure 1. One hundred measurement points were evenly distributed over the entire machined surface.

Results obtained from the dimensional accuracy measurements were then used to determine the process capability indices [1,2,3]. The values of Cp and Cpk were calculated using the equations:

(1) $$Cp=\frac{USL-LSL}{6*\sigma }=\frac{T}{6*\sigma }$$

(2) $$Cpk=\min\left\{\;\frac{USL-\bar{x}}{3*\sigma };\frac{\bar{x}-LSL}{3*\sigma }\;\right\}$$

where the process self-variability standard deviation *σ* is determined with the formula:

(3) $$\sigma =\frac{\bar{R}}{d_{2}}$$

where *R* is the range, and *d*_2_ is the statistical coefficient dependent on the group size.

The Pp and Ppk indices were calculated using the equations:

(4) $$Pp=\frac{USL-LSL}{6*s}=\frac{T}{6*s}$$

(5) $$Ppk=\min\left\{\;\frac{USL-\bar{x}}{3*s};\frac{\bar{x}-LSL}{3*s}\;\right\}$$

where the standard deviation *s* is calculated using the formula:

(6) $$s=\sqrt{\frac{1}{n-1}\overset{n}{\underset{i=1}{\sum }}{\left(x_{i}-\bar{x}\right)}^{2}}$$

Obtained results were analysed using Statistica 13 software.

## 3. Results and Discussion

### 3.1. Assessment of Data Distribution Normality

First of all, the normality of the distribution of dependent variables was verified, which is a prerequisite for determining process capability indices. For this purpose, the Shapiro–Wilk test was used, which is one of the most commonly used for the verification of the normal distribution of data. The *p*-value and W test results obtained for both tools with respect to the change in technological parameters are shown in Table 3.

**Figure 2:**
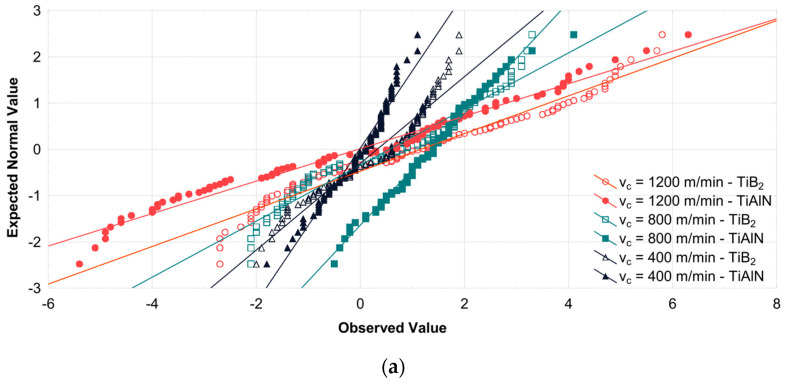

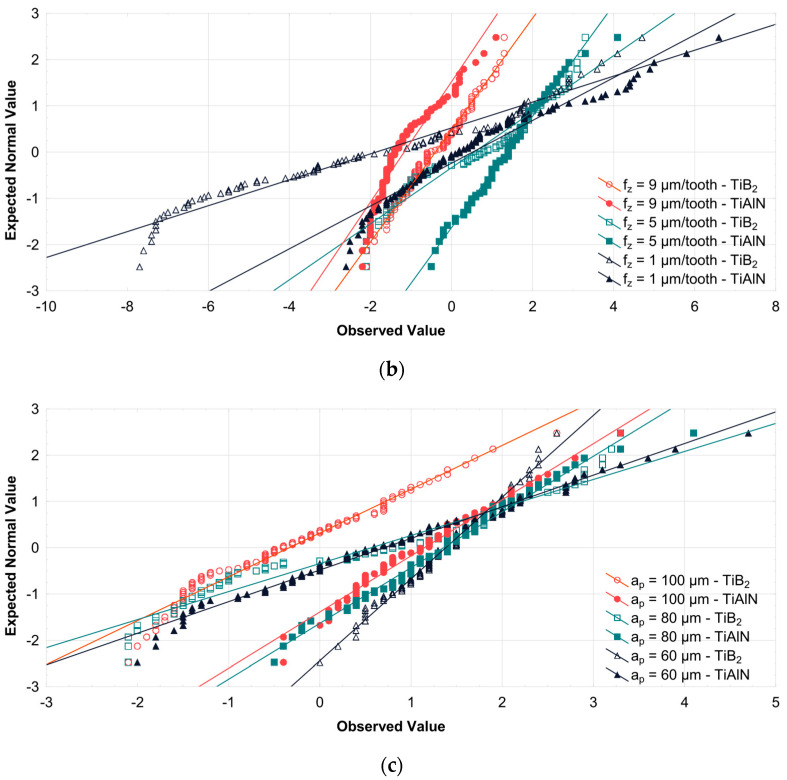
Assessment of data distribution normality using variable (**a**) cutting speed; (**b**) feed per tooth; (**c**) axial depth of cut.

Diagrams illustrating the normality of data obtained from a milling process conducted with variable technological parameters using both cutting tools are also shown in the form of charts in Figure 2.

In most cases, the obtained results did not meet the assumption of normal distribution, which made it impossible to determine the values of the process capability indices. Consequently, data with a non-normal distribution were subjected to the Johnson transformation in order to achieve the best fit of the distribution.

### 3.2. Determination of Process Capability Indices

After that, values of the indices Cp, Cpk and Pp, Ppk were calculated for the adopted tolerance T = 4 µm, evenly distributed with respect to the zero line. The indices values determined for both cutting tools depending on the variable technological parameters are listed in Table 4.

The high values of the Cp and Cpk indices confirm a small scatter of values for individual tool passes. Nevertheless, the significant differences between the values of Cp, Cpk and Pp, Ppk indicate the presence of within-group variability, i.e., within the results obtained from successive tool passes. Therefore, the use of Cp and Cpk for process control would lead to incorrect conclusions and overestimated process capability because the range of the results obtained for the entire process is much wider. For this reason, in a further part of this study, the focus was put on the Pp and Ppk indices.

**Figure 3:**
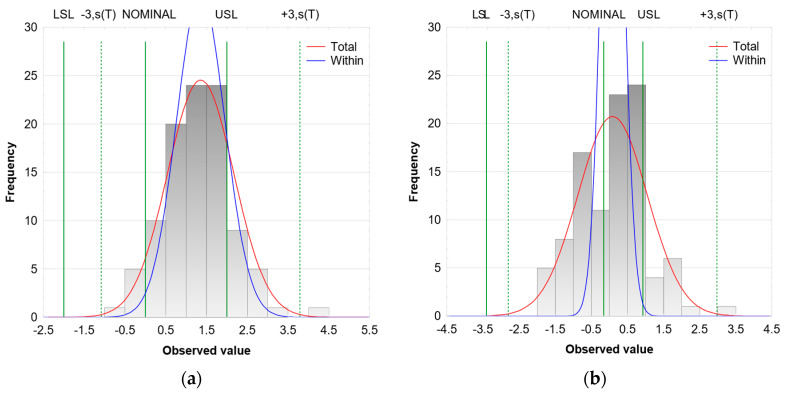
Distribution of the results obtained in a milling process conducted with (**a**) TiAlN-coated tool and (**b**) TiB_2_-coated tool and intermediate values of technological parameters.

Figure 3 also shows an example of the distribution of results obtained in a milling process conducted with intermediate values of technological parameters. It can be observed that the within variability (blue line) is reduced relative to the overall variability (red line), which reflects the differences between the indices. This is particularly evident in the results obtained for milling with the TiB_2_-coated tool.

**Figure 4:**
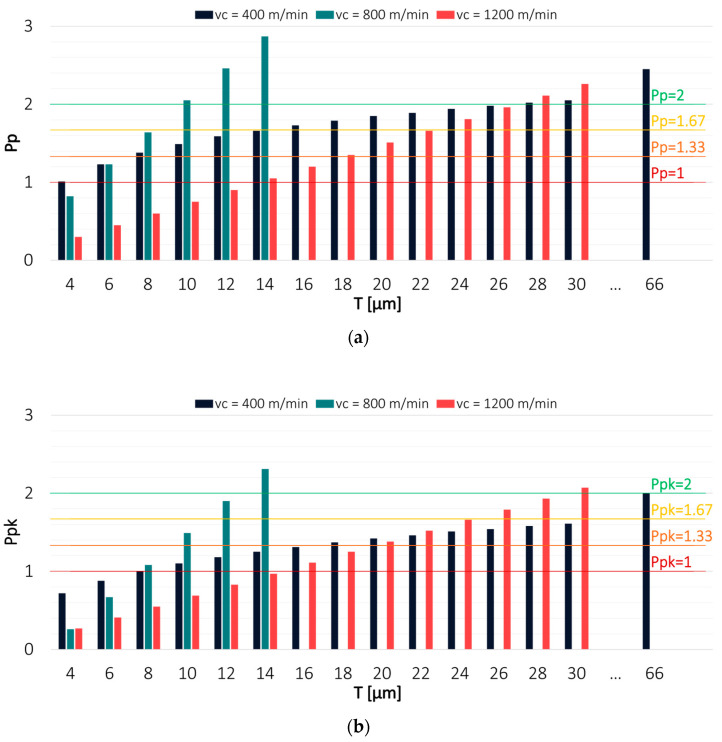
Values of (**a**) Pp and (**b**) Ppk for the results obtained in a milling process conducted with variable cutting speed and TiAlN-coated tool.

**Figure 5:**
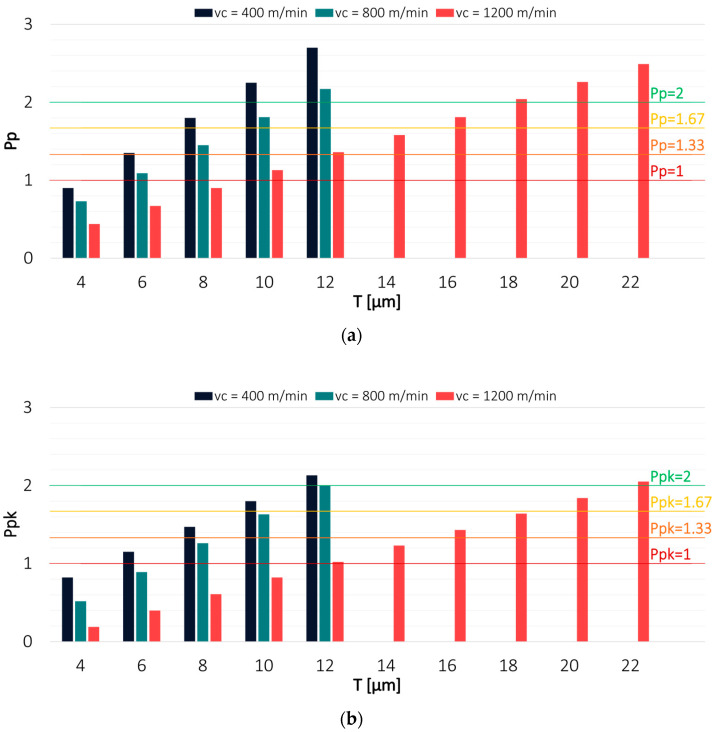
Values of (**a**) Pp and (**b**) Ppk for the results obtained in a milling process conducted with variable cutting speed and TiB2-coated tool.

Since the process capability indices depend on the assumed specification limits LSL and USL, Pp and Ppk values were determined for a tolerance range T that was changed every 2 µm. This approach was adopted to determine the tolerance at which it was possible to achieve particular levels of process capability. To facilitate the interpretation of the results, the most frequently used values of the capability indices are also marked on the charts as horizontal lines. The estimation was made for the results obtained in milling conducted with variable technological parameters: cutting speed v_c_, feed per tooth f_z_ and axial depth of cut a_p_. The values of Pp and Ppk indices for both tools in a milling process conducted with variable cutting speed v_c_ are shown in Figure 4 and Figure 5.

Regarding the TiAlN-coated tool, the highest capability was observed for a milling process conducted with an intermediate cutting speed value of v_c_ = 800 m/min, for which the Pp index value exceeded 2 already at T = 10 µm. Due to a lack of process centring, the Ppk value exceeded 2 at a tolerance of 14 µm. The lowest capability was observed for a milling process carried out with the lowest cutting speed of v_c_ = 400 m/min. To achieve a Pp index value of 2, the tolerance had to be increased to 30 µm, while in the case of the Ppk index, it had to be increased to as much as 66 µm.

An opposite relationship was observed for a milling process conducted using the TiB_2_-coated tool, where the highest process capability was obtained with the lowest cutting speed. The Pp and Ppk values were greater than 2 at a tolerance of 12 µm. A similar result was obtained when machining was conducted with v_c_ = 800 m/min. The worst process capability was observed for a milling process performed with the highest cutting speed v_c_ = 1200 m/min. However, the Pp and Ppk values exceeding 2 were achieved at T = 22 µm, which is a much better result than that obtained for the TiAlN-coated tool. The smaller differences between Pp and Ppk indicate that the results obtained with the TiB_2_-coated tool were also characterised by better centring. The cause of the differences in the obtained results may be due to different tool properties resulting from the application of the coating on the cutting edges of the end mill.

**Figure 6:**
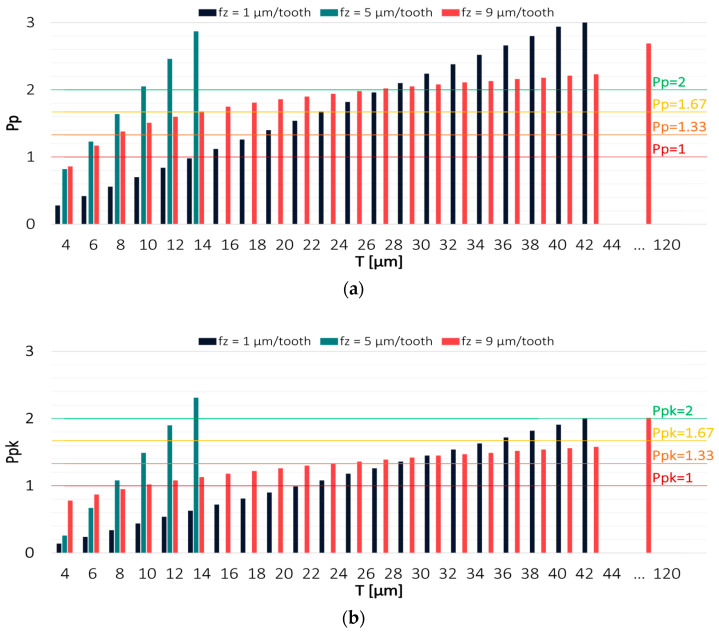
Values of (**a**) Pp and (**b**) Ppk for the results obtained in a milling process conducted with variable feed per tooth and TiAlN-coated tool.

**Figure 7:**
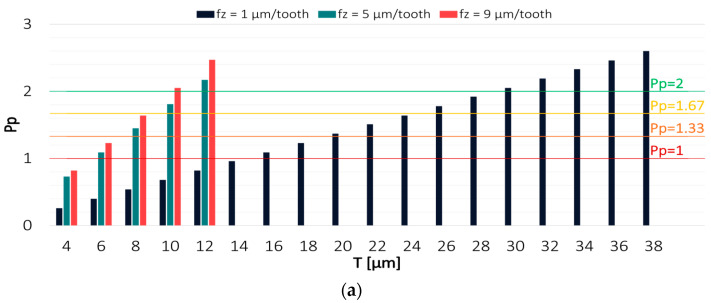

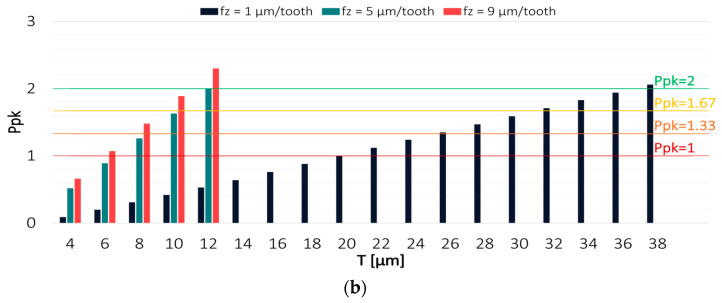
Values of (**a**) Pp and (**b**) Ppk for the results obtained in a milling process conducted with variable feed per tooth and TiB_2_-coated tool.

Figure 6 and Figure 7 show the values of Pp and Ppk indices for the results obtained from a milling process conducted with variable feed per tooth. The highest process capability was achieved when machining was carried out using the TiAlN-coated tool with intermediate values of technological parameters. Considerably worse results were obtained with the extreme values of feed per tooth. For the feed per tooth f_z_ = 1 µm/tooth, the Pp index exceeded a value of 2 at a tolerance of 30 µm; however, due to a relatively large displacement of the scatter field relative to the tolerance range, the Ppk index exceeded the desired level only at T = 44 µm. Significantly reduced centring and, at the same time, the lowest process capability were obtained in a milling process conducted with the highest feed per tooth value of f_z_ = 9 µm/tooth, where the Ppk index reached a value of 2 only at a tolerance of 120 µm, in spite of the fact that the Pp index already achieved this value at T = 20 µm.

Results obtained with the TiB_2_-coated tool were considerably less shifted from the expected value, and the effect of using variable technological parameters was the opposite of that observed for the TiAlN-coated tool. The highest process capability was obtained in a milling process conducted with the highest feed per tooth, where the values of both indices were above 2 already at a tolerance T = 12 µm. Similar results were obtained in a milling process conducted with an intermediate feed per tooth value of f_z_ = 5 µm/tooth. In contrast, the worst result was achieved in a milling process conducted with the lowest feed per tooth, for which the Pp index exceeded a value of 2 at a tolerance of 30 µm and the Ppk index at 38 µm. Nevertheless, this result is much better than that obtained for the TiAlN-coated tool. The ploughing phenomenon, which potentially affects the temperature in the cutting zone, causing thermal expansion of materials, may be of significant importance in this case.

**Figure 8:**
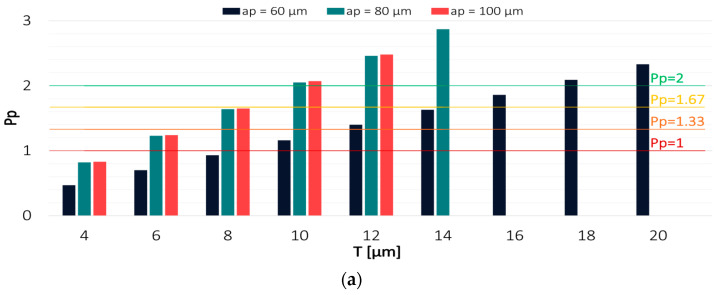

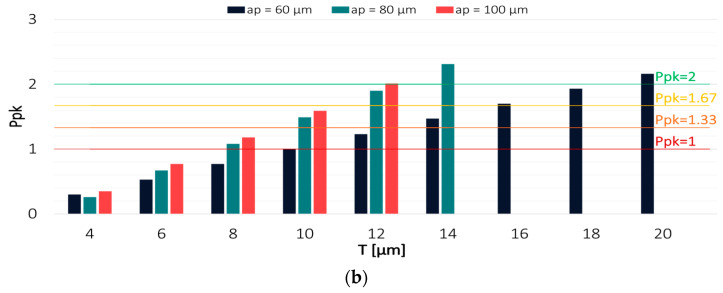
Values of (**a**) Pp and (**b**) Ppk for the results obtained in a milling process conducted with variable feed per tooth and TiAlN-coated tool.

**Figure 9:**
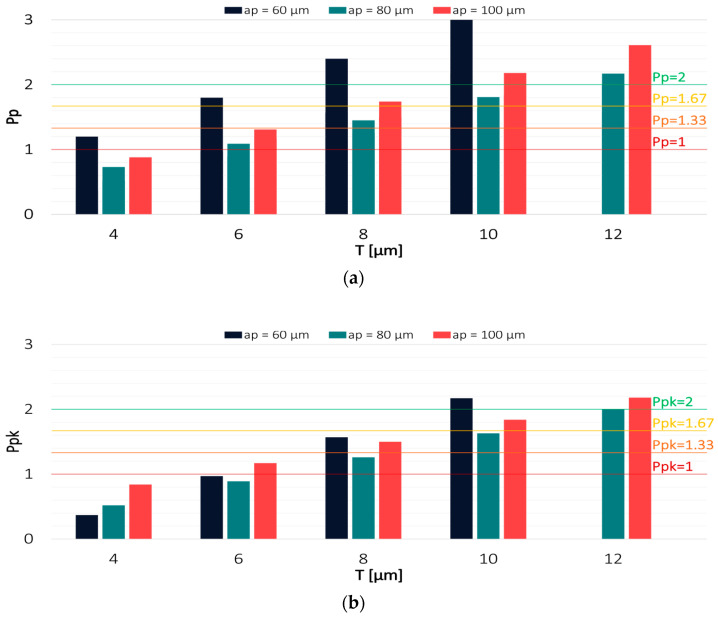
Values of (**a**) Pp and (**b**) Ppk for the results obtained in a milling process conducted with variable axial depth of cut and TiB_2_-coated tool.

Values of the Pp and Ppk indices for the results obtained in the milling process conducted with the variable axial depth of cut are shown in Figure 8 and Figure 9.

Among the analysed technological parameters, the variable axial depth of cut had the most negligible impact on the machining effect. With decreasing the axial depth of cut, the capability of the process conducted with the TiAlN-coated tool decreased, and the results increasingly shifted from the expected value. The highest process capability was achieved in milling with the axial depth of cut a_p_ = 100 µm, for which the Pp and Ppk indices reached a value of 2 at a tolerance of 10 µm and 12 µm, respectively. In contrast, the lowest process capability was achieved for the milling process conducted with a_p_ = 60 µm, where the Ppk value exceeded 2 only at T = 20 µm.

Regarding the TiB_2_-coated tool, the highest process capability was achieved when milling was conducted with the lowest depth of cut a_p_ = 60 µm. The highest values of Pp and Ppk were obtained with tolerances of 8 µm and 10 µm, respectively. For the depths of cut a_p_ = 80 µm and a_p_ = 100 µm, the tolerance had to be increased to 12 µm in order to achieve a Ppk value that would be greater than 2. This demonstrates that the variable axial depth of cut had even less effect on the results when the milling process was conducted with the TiB_2_-coated tool.

## 4. Conclusions

The results of this study and their analysis lead to the following main conclusions:

- The results were characterised by low within variability, which resulted in high values of Cp and Cpk. Failure to verify the variability between individual groups may lead to an overestimation of process capability.
- The change in technological parameters had a different effect on the results of the milling process depending on the cutting tool used due to the different properties of coated and uncoated tool cutting edges.
- The change of cutting speed and feed per tooth led to a considerably greater reduction in process capability when milling was conducted with a TiAlN-coated tool, caused probably by a temperature rise in the cutting zone.
- The change of axial depth of cut had a negligible impact on the machining effect, particularly when milling was conducted with a TiB_2_-coated tool.
- The milling process with the use of a TiAlN-coated tool was significantly less centred, particularly when machining was carried out with the lowest cutting speed and highest feed per tooth.
- The use of a TiB_2_-coated tool makes it possible to obtain much better machining results and, thus, the desired process capability with narrower tolerance range limits.

The results presented in this paper and their analysis have demonstrated that the selection of process capability indices for statistical process control is of vital importance. Although the Cp and Cpk indices are widely used, their improper selection may have serious negative effects on the manufacturing process, as incorrect result analysis may lead to the production of components that do not meet specific requirements, which leads to unnecessary costs.

The conducted research provided preliminary information on the achievable process capability of magnesium alloy precision milling. In particular, it was determined which factors determine the process capability the most. The aim of future research will be to investigate the reasons for these changes and attempt to process further improvement. It is also planned to conduct experiments with the use of cooling fluids to stabilise the temperature in the cutting zone, which presumably affects the resulting dimensional changes.

## Figures and Tables

**Table 1:** Chemical composition of AZ91D magnesium alloy.

Al	Mn	Zn	Cu	Others	Mg
2.5–3.5	0.1–0.2	0.7–1.3	0.05	0.04	Bal.

**Table 2:** Plan of experiments and tested values of technological parameters.

v_c_ [m/min]	f_z_ [µm/tooth]	a_p_ [µm]	a_e_ [mm]
400	5	80	14
800			
1200			
800	1	80	
	5		
	9		
800	5	60	
		80	
		100	

**Table 3:** Results of data normality distribution test.

Test Result	v_c_ [m/min]			f_z_ [µm/tooth]			a_p_ [µm]		
	400	800	1200	1	5	9	60	80	100
**TiAlN**									
***p*** **-value**	0.0032	0.3724	0.0490	0.0002	0.3724	0.0000	0.2432	0.3724	0.0998
**W**	0.9585	0.9860	0.9745	0.9398	0.9860	0.9033	0.9834	0.9860	0.9784
**TiB_2_**									
***p*** **-value**	0.0001	0.0001	0.0021	0.0011	0.0001	0.1590	0.5017	0.0001	0.0159
**W**	0.9296	0.9368	0.9561	0.9519	0.9368	0.9810	0.9879	0.9368	0.9681

**Table 4:** Process capability indices versus variable technological parameters and tool coating type.

Index	v_c_ [m/min]			f_z_ [µm/tooth]			a_p_ [µm]		
	400	800	1200	1	5	9	60	80	100
**TiAlN**									
**Cp**	1.48	1.12	0.92	0.63	1.12	0.76	1.16	1.12	1.05
**Pp**	1.01	0.82	0.30	0.28	0.82	0.66	0.47	0.82	0.83
**Cpk**	1.05	0.36	0.92	0.47	0.36	0.64	0.75	0.36	0.45
**Ppk**	0.72	0.26	0.27	0.14	0.26	0.56	0.30	0.26	0.35
**TiB_2_**									
**Cp**	2.15	2.42	1.68	1.15	2.42	1.17	1.57	2.42	1.58
**Pp**	0.90	0.73	0.44	0.26	0.73	0.82	1.20	0.73	0.87
**Cpk**	1.72	0.94	0.67	0.40	0.94	0.94	0.49	0.94	1.34
**Ppk**	0.82	0.52	0.19	0.09	0.52	0.66	0.37	0.52	0.83

## Data Availability

The raw/processed data required to reproduce these findings cannot be shared at this time as the data also form part of an ongoing study.
